# Mini-fluid challenge: how much fluid and what parameter to use?

**DOI:** 10.1186/cc12142

**Published:** 2013-03-19

**Authors:** B Geerts, RB De Wilde, JJ Maas, LP Aarts, JR Jansen

**Affiliations:** 1 Leiden University Medical Centre, Leiden, the Netherlands

## Introduction

The mini-fluid challenge is a widely used strategy to manage fluid loading in the ICU and OR. Although it might be a rational strategy, data on the mini-fluid challenge and its reliability are very limited. We investigated the value of changes in pulse contour cardiac output as a result of a mini-fluid challenge of 50 and 100 ml to predict fluid loading responsiveness.

## Methods

We measured the effects after the administration of 50, 100 and 500 ml bolus colloid infusions on CO (Modelflow (COm) and LiDCO (COli)), CVP and MAP in 21 patients on mechanical ventilation after elective cardiothoracic surgery. From the data we analysed the smallest volume that was predictive for the effects of 500 ml on cardiac output.

## Results

COli and COm increased after 50, 100 and 500 ml fluid loading. Best results are observed for changes in COm after 100 ml fluid loading (area under the ROC 0.86, 95% CI between 0.65 and 1.00). A change in Modelflow CO of at least 4.3% has a sensitivity of 67% and a specificity of 100% after 100 ml fluid loading. Sensitivity is 60% and specificity 83% for a similar cutoff in CO measured with the LiDCO device after 100 ml fluid loading. In our patient population, MAP and COli did not predict responsiveness with more accuracy than mathematical chance. See Figure 1.

## Conclusion

Changes in pulse contour CO can be used in a mini-fluid challenge to assess fluid responsiveness in our postcardiac surgery patients.

